# Recognition and management of early complications in CAR-T cell therapy and virus-specific T-lymphocyte therapy: a practical clinical reference

**DOI:** 10.3389/fimmu.2026.1884854

**Published:** 2026-08-28

**Authors:** Marek Ussowicz, Ewa Jakubczyk, Tomasz Wróbel, Anna Czyż

**Affiliations:** 1 Department of Pediatric Bone Marrow Transplantation, Oncology, and Hematology, Wroclaw Medical University, Wrocław, Poland; 2 Department of Pediatric Bone Marrow Transplantation, Oncology, and Hematology, University Clinical Hospital, Wrocław, Poland; 3 Department and Clinic of Hematology, Cellular Therapies, and Internal Medicine, Wroclaw Medical University, Wrocław, Poland

**Keywords:** CAR-T cell therapy, cytokine release syndrome (CRS), ICAHT, ICANS - immune effector cell-associated neurotoxicity syndrome, immunotherapy toxicity, tabelecleucel, virus-specific T lymphocytes

## Abstract

**Background:**

Cellular immunotherapies—including chimeric antigen receptor T-cell (CAR-T) therapies and adoptive transfer of virus-specific T lymphocytes (VSTs) — have transformed the treatment of refractory hematological malignancies and post-transplant infectious complications. Eight products are currently authorized by the European Medicines Agency, encompassing seven autologous CAR-T products targeting CD19 or BCMA and tabelecleucel (Ebvallo), the first approved allogeneic off-the-shelf EBV-specific T-cell product for EBV-positive post-transplant lymphoproliferative disease. Despite their clinical efficacy, both modalities carry distinct early toxicity profiles that differ from conventional cytotoxic chemotherapy.

**Objective:**

This review summarizes current recommendations for identifying, assessing, and treating early problems that can occur after CAR-T cell therapy and VST administration.

**Content:**

CAR-T-specific complications discussed include cytokine release syndrome (CRS), immune effector cell-associated neurotoxicity syndrome (ICANS), immune effector cell-associated hematotoxicity (ICAHT), and immune effector cell-associated HLH-like syndrome (IEC-HS). CRS is graded by ASTCT consensus criteria and managed with tocilizumab as first-line pharmacological therapy; steroid-refractory ICANS is most commonly treated with high-dose intravenous anakinra (up to 12 mg/kg/day) which is currently the most studied second-line option, although the supporting evidence remains largely observational. ICAHT is classified using the validated EHA/EBMT grading framework, separating early (day 0–30) and late (post-day 30) neutropenia by depth and duration, with management escalating from prophylactic G-CSF through hematopoietic cell boost to allogeneic HSCT as the ultimate option. For VSTs, the principal early complications are tumor flare reaction (in approximately 20% of tabelecleucel recipients), GVHD (below 5% with enriched products), acute infusion reactions, and low-grade CRS-like cytokine release. We summarize a differential diagnosis of overlapping syndromes, pediatric-specific adaptations, ICU escalation criteria, and a clinical monitoring schedule.

**Conclusions:**

Internationally validated criteria grade the early complications of cellular immune effector therapies. Prompt recognition, early pharmacological intervention, and monitoring are essential to minimize non-relapse mortality. Expanding real-world experience and the integration of pre-treatment risk stratification tools will continue to refine evidence-based practice in this rapidly evolving field, ultimately leading to improved patient outcomes and reduced non-relapse mortality rates.

## 1 Introduction

The field of adoptive cellular immunotherapy has witnessed unprecedented advances, with the regulatory approval of multiple chimeric antigen receptor (CAR) T-cell products and the first allogeneic virus-specific T-cell (VST) product for clinical use. These therapies offer treatment options in refractory hematological malignancies and infectious complications. However, their administration is accompanied by a distinct and sometimes life-threatening toxicity profile that differs fundamentally from that of conventional cytotoxic chemotherapy, which can include severe side effects such as organ damage, increased risk of infections, and prolonged recovery times.

CAR-T cell therapy directs genetically modified T cells expressing synthetic antigen receptors to recognize and eliminate tumor cells bearing specific surface antigens (most commonly CD19 or BCMA). The consequent immune activation is potent but carries the risk of uncontrolled systemic cytokine release, neurological toxicity, prolonged cytopenias, and infectious complications.

VSTs represent a complementary cellular approach in which T cells with specificity for viral antigens—such as Epstein-Barr virus (EBV), cytomegalovirus (CMV), adenovirus (AdV), or BK virus (BKV)—are generated ex vivo and infused to restore antiviral immunity in immunocompromised transplant recipients (1). VSTs generally carry a more favorable toxicity profile than CAR-T cells, but specific early complications—particularly tumor flare reactions, GVHD, and acute infusion reactions — require specific management (2–4).

The EMA marketing authorization of tabelecleucel (Ebvallo) on 16 December 2022 marked a milestone as the first approved off-the-shelf, partially HLA-matched EBV-specific T-cell therapy for relapsed/refractory EBV-positive post-transplant lymphoproliferative disease (EBV+ PTLD) in adults and children ≥ 2 years of age (4). Tabelecleucel consists of allogeneic EBV-specific T cells derived from healthy donors, sensitized ex vivo by co-culture with donor B cells infected with EBV (lymphoblastoid cell lines), yielding a product partially HLA-matched to the recipient.

The EBMT/EHA CAR-T Cell Handbook and ASTCT consensus criteria provide the current reference framework for grading and management of cellular therapy complications across European transplant centers, accompanied by EBMT/JACIE practice recommendations (5, 6). Because CAR-T toxicities are characterized by large prospective trials and internationally validated grading systems, whereas VST safety data derive predominantly from smaller cohorts and, for the single approved product, regulatory documentation, the two modalities are covered at differing levels of evidence. We make this disparity explicit and summarize knowledge to aid orientation.

This article is a narrative, practice-oriented review rather than a systematic review. We based it on a defined set of international reference sources—the EBMT/EHA CAR-T Cell Handbook, the ASTCT consensus grading criteria, the EHA/EBMT ICAHT consensus, EBMT-JACIE best-practice recommendations, ASCO/IDSA and NCCN guidance, and EMA product information (EPAR/SmPC)—supplemented by targeted PubMed searches for pivotal registration trials and key real-world cohorts published up to February 2026. The text states that recommendations rely on expert consensus, retrospective data, or institutional practice instead of prospective evidence (4, 5, 7).

## 2 Regulatory landscape and approved cellular therapy products

As of February 2026, eight cellular therapy products are authorized by the EMA for hematological indications. The first seven are autologous CAR-T cell products; tabelecleucel (Ebvallo) is the first allogeneic off-the-shelf VST product.

**Table 1** summarizes the approved products, their targets, and major toxicity rates, drawing on EMA EPAR documentation and key pivotal trial publications (4, 8–13).

**Table 1:** EMA-authorized cellular therapy products.

Product	Target/indication	CRS (any grades)	ICANS (any grade)	EMA Approval	Source
Tisagenlecleucel (Kymriah)	CD19; B-ALL, LBCL, FL	77%; 20–23% ≥ 3	21–40%; 13% G ≥ 3	2018	JULIET trial (9) ELIANA trial (14)
Axicabtagene ciloleucel (Yescarta)	CD19; LBCL, FL, MZL	93%; 9–11% G ≥ 3	64%; 28–32% G≥3	2018	ZUMA trial (8)
Lisocabtagene maraleucel (Breyanzi)	CD19; LBCL, FL	42–49%; 1–2% G ≥ 3	27–30%; 10% G≥3	2022	TRANSCEND NHL 001 (15)
Brexucabtagene autoleucel (Tecartus)	CD19; MCL, ALL	91%; 11% G ≥ 3	69%; 31% G≥3	2020	ZUMA-2 (10) ZUMA-3 trial (16)
Obecabtagene autoleucel (Aucaytzyl)	CD19; r/r B-ALL (≥26 years)	68.5%; 2.4% G ≥ 3	22.8%; 7.1% G ≥ 3	2025	FELIX trial (13)
Idecabtagene vicleucel (Abecma)	BCMA: Multiple Myeloma	84%; 5% G≥3	18%; 3% G≥3	2021	KarMMa trial (17)
Ciltacabtagene autoleucel (Carvykti)	BCMA: Multiple Myeloma	95%; 4–5% G≥3	23–26%; 10% G≥3	2022	CARTITUDE-1 trial (18)
Tabelecleucel (Ebvallo)	EBV-VST (allogeneic); EBV+ PTLD (r/r); ≥2 years	N/A – TFR 20%; CRS-like < 5%	No ICANS; GVHD < 5%	2022	EBVOLVE (19) ALLELE (20)

Rates derive from heterogeneous pivotal trials and EMA EPAR documentation and are not directly comparable across products; per-product sources are cited in the final column.

B-ALL, B-cell acute lymphoblastic leukemia; BCMA, B-cell maturation antigen; FL, follicular lymphoma; LBCL, large B-cell lymphoma; MCL, mantle cell lymphoma; MZL, marginal zone lymphoma; TFR, tumor flare reaction.

All CAR-T products are classified as Advanced Therapy Medicinal Products (ATMPs) under EU Regulation 1394/2007. Tabelecleucel additionally holds orphan designation (EU/3/16/1627) and was approved under exceptional circumstances due to the rarity of EBV+ PTLD, with ongoing post-authorization study obligations (EBVOLVE study) (19). It was granted PRIME (Priority Medicines) designation, reflecting its potential to address unmet medical need.

## 3 Overview of early complications

Early complications of cellular therapies typically manifest within the first 7–30 days post-infusion, though certain toxicities—particularly late-onset ICANS and ICAHT—can present up to 6–8 weeks after CAR-T infusion (21–23).

### 3.1 CAR-T cell therapy: pathophysiology of early toxicity

Following CAR-T cell infusion and lymphodepletion conditioning, administered CAR-T cells encounter target antigen-expressing tumor cells. Recognition leads to T-cell activation, rapid *in vivo* expansion, and secretion of pro-inflammatory cytokines, including interleukin-6 (IL-6), interferon-gamma (IFN-γ), interleukin-1 (IL-1), and tumor necrosis factor-alpha (TNF-α) (21). Macrophage activation amplifies this cytokine cascade, leading to the multi-systemic inflammatory syndrome known as CRS. The inflammatory response further disrupts the blood–brain barrier through endothelial activation and VEGF-mediated permeability, precipitating ICANS (22, 24, 25). Bone marrow suppression occurs via persistent immune activation of CD8+ T cells and elevated IFN-γ in the marrow microenvironment, resulting in ICAHT (26).

Peak cytokine levels typically occur within 7–14 days of infusion and correlate with CAR-T *in vivo* expansion kinetics (27). Higher tumor burden at the time of infusion, lymphodepletion with fludarabine/cyclophosphamide, elevated pre-treatment inflammatory markers, and a greater number of prior therapy lines are consistently identified as risk factors for severe toxicity (26, 28).

### 3.2 Virus-specific T lymphocytes: pathophysiology of early toxicity

VSTs are associated with a more favorable early safety profile compared with CAR-T cells (1). The main early risks relate to alloreactivity in donor-derived or partially HLA-matched third-party products leading to GVHD; immune activation at tumor sites causing tumor flare reactions; acute infusion reactions, partly attributable to cryoprotectants such as DMSO; and very rarely, CRS-like cytokine release secondary to rapid T-cell expansion (1–3). The low alloreactivity of VSTs has been demonstrated in multiple clinical studies, with GVHD frequencies consistently below 5% for enriched products.

## 4 Cytokine release syndrome

### 4.1 Definition and incidence

Cytokine release syndrome (CRS) is the most common early complication of CAR-T cell therapy, occurring across all approved products at rates of 37–95% for any grade (21). CRS is defined as a systemic inflammatory response caused by rapid and massive cytokine release from immune effector cells engaged in an immune reaction. The ASTCT consensus criteria require fever ≥38 °C as the cardinal feature of any-grade CRS, except in patients who have received antipyretics or immunosuppressants prior to grading (6).

The frequency of severe (Grade ≥3) CRS varies considerably by product: axicabtagene ciloleucel exhibits higher CRS rates (9–11% Grade ≥3) compared with lisocabtagene maraleucel (1–2% Grade ≥3), reflecting differences in costimulatory domain (CD28 vs. 4-1BB) and lymphodepletion conditioning (8). In BCMA-directed products, CRS rates are high in any-grade terms (84–95%), but severe events are relatively infrequent (4–5%) (27).

Importantly, GM-CSF should be avoided in the setting of CAR-T cell therapy due to the risk of augmenting macrophage-driven cytokine production (27).

### 4.2 Clinical presentation

CRS typically manifests within 1–14 days post-infusion, with a median onset of approximately 2–3 days for CD19-directed products and 5–8 days for BCMA-directed products. Initial symptoms are non-specific and closely resemble infectious illness (27).

Laboratory findings associated with CRS include markedly elevated CRP (>200 mg/L), hyperferritinemia, elevated LDH, hyponatremia, coagulopathy with elevated D-dimers, and elevated troponin in cardiac involvement (27). Procalcitonin has limited diagnostic value in neutropenic patients, though levels below 0.4 ng/mL in non-neutropenic patients suggest absence of bacterial infection. Serum IL-6 is the cytokine most directly implicated in CRS pathophysiology, and its concentration broadly correlates with CRS severity and with CAR-T expansion. Its utility as a routine bedside biomarker is nevertheless limited: with turnaround times unsuited to real-time grading, no prospectively validated threshold guides intervention, and IL-6-receptor blockade with tocilizumab abolishes receptor-mediated clearance, producing a paradoxical rise in circulating IL-6 that renders post-treatment values uninterpretable as a measure of inflammatory activity. For these reasons, serial CRP and ferritin remain the more practical day-to-day markers, with IL-6 reserved for research or selected diagnostic situations.

Concurrent tumor lysis syndrome may occur in patients with high disease burden (27).

CRS symptoms must be differentiated from septic shock and bacterial infection. An infectious workup, including blood cultures, urinalysis, chest imaging, and relevant viral PCR testing (CMV, EBV, respiratory viruses), must be initiated simultaneously with CRS management in all febrile patients post-infusion. Empirical broad-spectrum antibacterial therapy should never be delayed pending cytokine results (5, 27, 29).

### 4.3 ASTCT consensus CRS grading criteria

The ASTCT 2019 consensus grading system is the internationally accepted tool for CRS severity assessment, adopted by EBMT, ASCO, NCCN, and JACIE guidelines (6). Grade is determined primarily by hemodynamic and oxygen requirement parameters (**Table 2**).

**Table 2:** ASTCT consensus grading and management of cytokine release syndrome.

Grade	Hypotension	Hypoxia
1	None	None
2	Not requiring vasopressors	Requiring low-flow nasal cannula (≤6 L/min) or blow-by
3	Requiring one vasopressor, with or without vasopressin	Requiring high-flow nasal cannula (>6 L/min), facemask, non-rebreather, or Venturi mask
4	Requiring multiple vasopressors (excluding vasopressin)	Requiring positive-pressure ventilation (CPAP, BiPAP, intubation/mechanical ventilation)

Fever (≥38 °C) is required for grade 1 and is a prerequisite for entry into grading; once tocilizumab or steroids have been given, fever is no longer required to grade subsequent severity. CRS grade is determined by the more severe of the hypotension or hypoxia parameters; organ toxicities are graded separately per CTCAE.

### 4.4 CRS management

**Figure 1:**
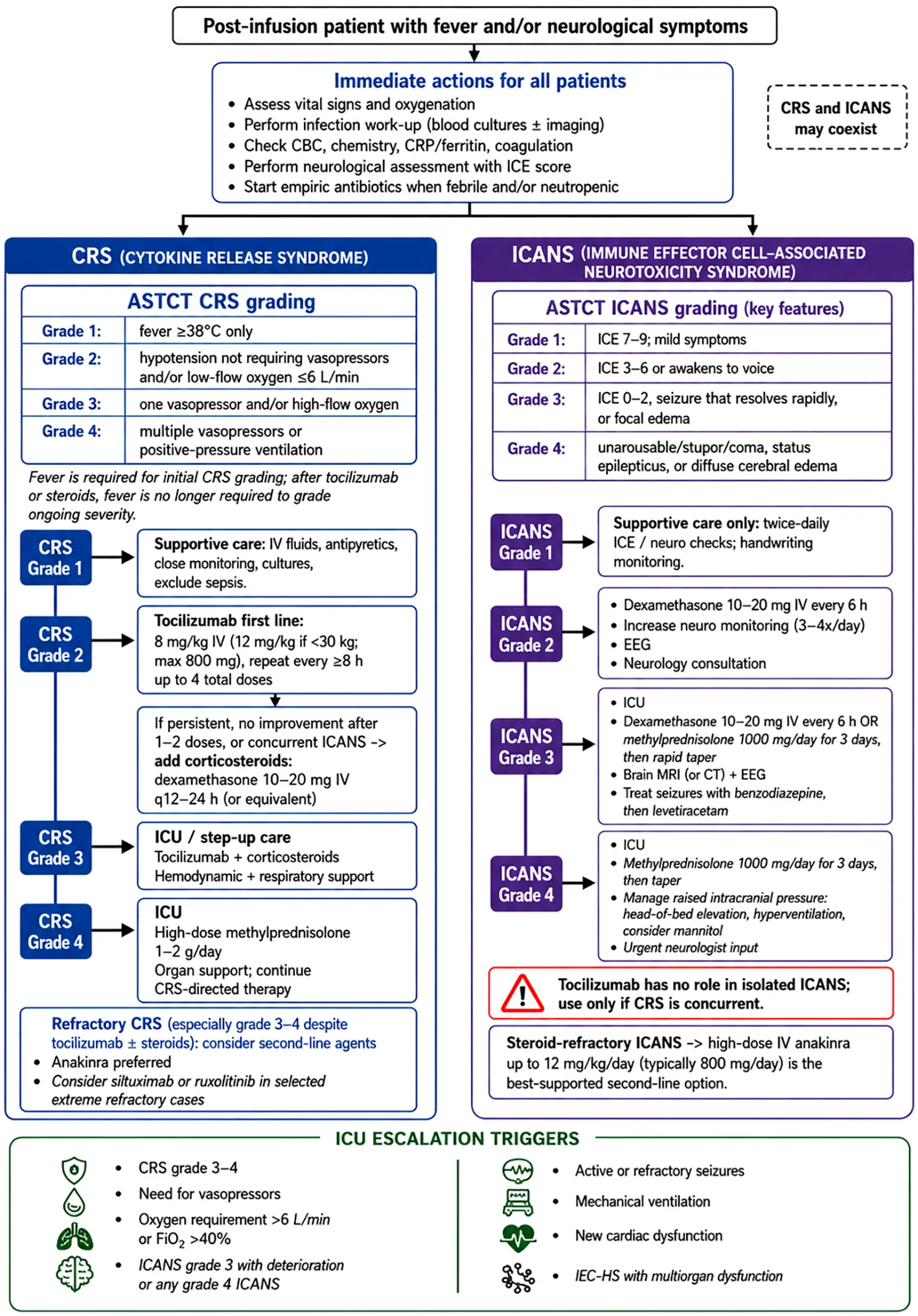
Management algorithm for CRS and ICANS after CAR-T therapy.

The treatment of CRS and ICANS is summarized in the **Figure 1**.

Tocilizumab, an anti-IL-6 receptor monoclonal antibody, is EMA- and FDA-approved for the treatment of Grade ≥2 CRS following CAR-T cell therapy (21). The recommended dose is 8 mg/kg (12 mg/kg for patients weighing less than 30 kg) IV over 60 minutes (maximum 800 mg), with up to 4 doses permitted at intervals of at least 8 hours (30). All major guidelines recommend tocilizumab as first-line pharmacological intervention for CRS requiring treatment (5, 7, 21). If no clinical improvement in the signs and symptoms of CRS occurs after the first dose, up to three additional doses may be administered, for a maximum of four doses per CRS episode, with a minimum interval of eight hours between consecutive doses. This dosing limit is specified in both the EMA Summary of Product Characteristics for tocilizumab (RoActemra) and the FDA-approved prescribing information for tocilizumab (Actemra), and derives from the regulatory approval dataset in which a median of one dose (range, 1–4) was administered across the pivotal retrospective analysis of 51 patients with severe or life-threatening CAR-T-induced CRS (31). Clinicians should be aware that tocilizumab itself alters subsequent laboratory monitoring. IL-6-receptor blockade suppresses the CRP response within 24–48 hours irrespective of whether an underlying infection is controlled; a falling CRP after tocilizumab therefore does not exclude evolving bacterial infection. Ferritin is comparatively less affected and, together with blood cultures and clinical assessment, should be relied upon when infection is suspected.

Importantly, short courses of corticosteroids do not appear to attenuate CAR-T anti-tumor efficacy based on contemporary data, and their use should not be withheld in clinically significant CRS (27). Prophylactic use of tocilizumab is not routinely recommended, as IL-6 receptor blockade leads to increased circulating IL-6 levels, which may cross the blood–brain barrier and has been associated with a higher risk or severity of ICANS (32).

For CRS refractory to 1–2 doses of tocilizumab, corticosteroids (dexamethasone 10–20 mg IV q12–24 h or methylprednisolone equivalent) should be initiated (5). In Grade 4 or tocilizumab-refractory Grade 3 CRS, high-dose methylprednisolone 1–2 g/day may be required (7, 21). Anakinra (IL-1 receptor antagonist) may be considered as first choice for severe CRS refractory to anti-IL-6 therapy and high-dose corticosteroids; limited evidence also exists for siltuximab and ruxolitinib in extreme refractory cases (21, 33).

The evidence supporting these agents varies and should be evaluated carefully; no randomized controlled trial was conducted for established CRS. Tocilizumab is regulatorily approved and uniformly recommended as first-line pharmacological therapy for grade >=2 CRS, and corticosteroids are guideline-supported for tocilizumab-refractory CRS or concurrent ICANS. In contrast, anakinra, siltuximab, and ruxolitinib are reserved for refractory cases and are supported predominantly by retrospective case series and expert opinions rather than prospective trial data.

## 5 Immune effector cell-associated neurotoxicity syndrome

### 5.1 Definition and incidence

Immune effector cell-associated neurotoxicity syndrome (ICANS) is defined as a disorder characterized by a pathological process involving the central nervous system (CNS) following any immunotherapy that results in activation or engagement of endogenous or infused T cells and/or other immune effector cells (6). Previously termed CAR-T-related encephalopathy syndrome (CRES), ICANS occurs in 20–60% of all CAR-T recipients, with severe (Grade ≥3) events in 3–30% (27). Incidence varies markedly by product: highest with axicabtagene ciloleucel (64% any grade; Grade ≥3 28–32%), and substantially lower with lisocabtagene maraleucel (27–30%; Grade ≥3 – 5%10%) (5, 27).

### 5.2 Pathophysiology

The precise mechanism of ICANS remains incompletely understood (22). Evidence supports that systemic cytokine release — particularly IL-6, IL-1, and IFN-γ — causes endothelial activation and increased vascular permeability, leading to disruption of the blood–brain barrier (BBB). Elevated CAR-T cells and cytokines in the CSF have been identified in severe ICANS, suggesting direct CNS involvement. ICANS may occur concurrently with, after resolution of, or — in approximately 10% of cases — more than 3 weeks after CRS (27).

There have been rare reports of loss of oligodendroglial lineage cells and multiple areas of demyelinating leukoencephalopathy after CD19 CAR-T therapy 28.

### 5.3 Clinical presentation and diagnosis

Onset of ICANS occurs on average approximately 5 days following CAR-T infusion. Clinical features are variable and typically progress through stages:

- Early/mild: Word-finding difficulty (aphasia), dysgraphia (handwriting deterioration), tremor, mild confusion—handwriting changes are an important early hallmark (27, 30)
- Moderate: Expressive and receptive aphasia, agitation, lethargy, impaired short-term memory
- Severe: Obtundation, stupor, tonic-clonic seizures, status epilepticus, cerebral edema, coma

The Immune Effector Cell-Associated Encephalopathy (ICE) score is the validated 10-point screening tool for ICANS, assessing orientation, naming, following commands, writing, and attention. It is used together with an assessment of consciousness level, seizure evaluation, and motor function to determine the overall ICANS grade. ICE screening every 8–12 hours is recommended during the acute post-infusion period.

ICANS is a clinical diagnosis. Brain MRI and CSF examination are rarely diagnostic of ICANS itself but are important to exclude alternative diagnoses (CNS infection, malignant leptomeningeal disease, progressive multifocal leukoencephalopathy, and cerebrovascular events). EEG should be obtained for any grade ≥2 ICANS to detect non-convulsive seizures (34).

### 5.4 ICANS grading and management

ICANS is graded on a four-point scale that integrates the ICE score, level of consciousness, seizure activity, motor findings, and signs of raised intracranial pressure, with the overall grade set by the most severe parameter (**Table 3**). Management escalates in parallel with grade, but several interventions warrant earlier consideration than the grade thresholds alone might suggest. Neurology consultation and an EEG to exclude non-convulsive status epilepticus should be considered from grade 2, and brain MRI should be obtained whenever focal signs, seizures, or failure to improve raise the possibility of a structural or alternative cause. Transfer to intensive care is appropriate from grade 3. Corticosteroids are the pharmacological mainstay across grades 2–4—dexamethasone at lower grades, escalating to high-dose methylprednisolone for grade 3–4 or progressive disease. The measures listed for raised intracranial pressure at grade 4 (head-of-bed elevation, controlled hyperventilation, and hyperosmolar therapy with mannitol or hypertonic saline) are neurocritical-care interventions and should be delivered together with neurology and intensive-care specialists rather than applied as routine bedside instructions.

**Table 3:** Grading and management of immune effector cell-associated neurotoxicity syndrome (ICANS).

Overall ICANS grade	ICE score	Depressed level of consciousness	Seizure	Motor findings	Elevated ICP/cerebral edema	Management
Grade 1	7-9	Awakens spontaneously	N/A	N/A	N/A	Supportive care only. Twice-daily neurocognitive assessment using ICE score
Grade 2	3-6	Awakens to a voice	N/A	N/A	N/A	Supportive care as per Grade 1. Administer dexamethasone 10–20 mg IV every 6 hours. Increase ICE score neuromonitoring (e.g., 3-4x/d). Consider EEG to exclude non-convulsive seizures and request neurology consultation
Grade 3	0-2	Awakens only to tactile stimulus	Any clinical seizure, focal or generalized, that resolves rapidly or non-convulsive seizures on EEG that resolve with intervention	N/A	Focal/local edema on neuroimaging	Transfer the patient to the intensive-care unit (ICU) and have assessment by a neurologist. Administer dexamethasone 10–20 mg IV every 6 hours, or methylprednisolone 1000 mg/day for 3 days until improvement to Grade 1 and then rapid taper (e.g., 250 mg–125 mg–60 mg for 1 day each). Treat seizures with lorazepam IV or other benzodiazepines as needed, followed by loading with levetiracetam. Obtain brain MRI (or CT if MRI is not feasible) and EEG to exclude alternative pathology and non-convulsive seizures.
Grade 4	0 (patient is unarousable and unable to perform ICE)	Patient is unarousable or requires vigorous or repetitive tactile stimuli to arouse. Stupor or coma	Life-threatening prolonged seizure (>5 min); or repetitive clinical or electrical seizures without return to baseline in between	Deep focal motor weakness such as hemiparesis or paraparesis	Diffuse cerebral edema on neuroimaging, decerebrate or decorticate posturing, cranial nerve VI palsy, papilledema, or Cushing’s triad	Management as per Grade 3. Administer methylprednisolone 1000 mg/day for 3 days, then taper at 250 mg every 12 hrs for 2 days, then 125 mg every 12 hrs for 2 days, then 60 mg every 12 hrs for 2 days. For convulsive status epilepticus, seek urgent advice from a neurologist. For management of raised intracranial pressure, elevate the head of the bed, set ventilatory settings to hyperventilation, and consider hyperosmolar therapy with mannitol.

Steroid-Refractory ICANS (srICANS) is defined as failure to improve Grade (3/)4 ICANS after 3 days of high-dose steroids. Evidence guiding its management remains limited, but several second-line options exist.

- Anakinra (IL-1 receptor antagonist) is the best-supported option, with an overall response rate of 77%. High-dose IV anakinra (up to 12 mg/kg/day, typically 800 mg/day) is preferred over low-dose regimens (100–200 mg/day), as it achieved faster ICANS resolution within 7 days and lower 28-day treatment-related mortality (35).
- Intrathecal triple therapy (hydrocortisone 50 mg + methotrexate 12 mg + cytarabine 50 mg) offers a CNS-targeted alternative by directly addressing CAR-T activity in the central nervous system. It was well tolerated and produced rapid, sustained improvement in an srICANS cohort (36, 37).
- Other agents—siltuximab (IL-6 inhibitor), ruxolitinib (JAK inhibitor), and dasatinib (tyrosine kinase inhibitor) — show preclinical promise but lack sufficient clinical evidence to support routine use.

High-dose IV anakinra is currently the most studied second-line agent for steroid-refractory ICANS, with reported overall response rates around 77%, but this evidence is largely observational and derived from retrospective cohorts; a randomized trial of prophylactic anakinra is ongoing. It may reasonably be considered a preferred second-line option, with intrathecal triple therapy as a reasonable CNS-directed alternative pending prospective confirmation.

## 6 Immune effector cell-associated hematotoxicity

### 6.1 Definition and clinical significance

Hematological toxicity is the most common adverse event after CAR-T cell therapy, representing the most frequent Grade ≥3 toxicity in real-world cohorts (23, 28). Cytopenias can be profound and prolonged and predispose patients to severe infectious complications, transfusion dependence, and high non-relapse mortality (NRM). In a real-world cohort of 549 patients (MM, LBCL, MCL), severe ICAHT (Grade ≥3) was independently associated with increased infection incidence, transfusion dependence, and significantly inferior progression-free and overall survival (28).

To address this unique pattern of hematotoxicity, the European Hematology Association (EHA) and EBMT convened an international panel of 36 experts and published consensus grading and management recommendations in Blood (26). The term “ICAHT” was introduced to capture the biphasic and/or delayed course of post-CAR-T cytopenias: an early aplasia phase attributable to lymphodepletion and direct marrow cytokine effects, and a late recovery phase complicated by persistent immune activation. Single-cell RNA sequencing of post-CAR-T bone marrow samples has revealed persistently expanded CD8+ IFN-γ-expressing T cells, suggesting that chronic marrow exposure may suppress hematopoiesis in late ICAHT (28, 38).

### 6.2 Risk factors and pre-infusion stratification

Pre-infusion risk factors for severe ICAHT include higher baseline bone marrow tumor burden, lower pre-treatment blood counts, elevated baseline inflammatory markers (CRP, ferritin), a higher number of prior therapy lines, and more severe post-CAR-T CRS or ICANS. The CAR-HEMATOTOX score is a validated pre-infusion risk stratification tool designed to identify patients at high risk of clinically significant hematotoxicity following CAR-T cell therapy (26, 39, 40). The score integrates five parameters assessed prior to lymphodepletion (day −5): platelet count, absolute neutrophil count, hemoglobin concentration, C-reactive protein, and serum ferritin. Individual parameters are scored on an ordinal scale of 0–1–2, with platelet count and ferritin scored 0–2 and the remaining parameters scored 0–1, yielding a maximum total of 8 points. Patients are classified as low risk (score 0–1) or high risk (score ≥2), enabling early identification of individuals at elevated likelihood of developing post-CAR-T cytopenias. In the original derivation cohort of adult patients with relapsed/refractory large B-cell lymphoma treated with tisagenlecleucel or axicabtagene ciloleucel following fludarabine/cyclophosphamide lymphodepletion, high CAR-HEMATOTOX scores correlated significantly with the development of severe neutropenia, thrombocytopenia, and anemia. Importantly, the score demonstrates high sensitivity and high negative predictive value, but relatively low specificity, positioning it appropriately as a screening instrument to flag patients who may develop hematological complications rather than as a tool to exclude patients from CAR-T eligibility. In clinical practice, a high CAR-HEMATOTOX score should prompt proactive risk-adapted interventions including prophylactic G-CSF administration, antibacterial and antifungal prophylaxis, and intensified hematological monitoring.

### 6.3 ICAHT grading

The EHA/EBMT grading system classifies ICAHT separately for early (days 0–30) and late (after day 30) cytopenias, using depth and duration of neutropenia as the primary determinants (**Table 4**) (23). The system has been prospectively validated across multiple cohorts including BCMA- and CD19-directed products in multiple myeloma, large B-cell lymphoma, and mantle cell lymphoma (28).

**Table 4:** EHA/EBMT consensus grading of immune effector cell-associated hematotoxicity (ICAHT).

Complication		Grade 1	Grade 2	Grade 3	Grade 4
Early ICAHT (days 0-30)	Severe Neutropenia	1–6 days	7–13 days	≥14 days	Never above ANC 500/µL
	(ANC <0.5 G/L)				
	Profound Neutropenia	-	-	≥7 days	≥14 days
	(ANC <0.1 G/L)				
Late ICAHT (after day 30)	ANC	<1500/µL	<1000/µL	<500/µL	<100/µL

### 6.4 Management of ICAHT

#### 6.4.1 Risk stratification and early prophylaxis (grade I)

Even at the earliest, mildest stage, the algorithm calls for risk assessment using the CAR-HEMATOTOX score. Patients identified as high-risk may receive prophylactic G-CSF starting from approximately day +2. Although G-CSF was historically withheld in the early post-infusion period over a theoretical risk of aggravating cytokine-mediated toxicity, contemporary cohorts have not confirmed this concern: prophylactic or early (day +2 to +5) G-CSF has been associated with faster neutrophil recovery and a significant reduction in febrile neutropenia without a demonstrable increase in grade >=2 CRS or ICANS, and risk-adapted use is endorsed by the EHA/EBMT ICAHT consensus (26, 41). The optimal timing nonetheless remains incompletely defined and should be individualized, with documented severe infection prompting a case-by-case risk-benefit assessment. High-risk patients should additionally receive the broader prophylaxis outlined in Section 8: antibacterial prophylaxis guided by local multidrug-resistant organism epidemiology, mold-active antifungal prophylaxis, antiviral and Pneumocystis jirovecii prophylaxis, and immunoglobulin replacement for IgG < 4 g/L.

#### 6.4.2 Therapeutic G-CSF for persistent neutropenia

For patients with persistent neutropenia meeting Grade II–IV criteria, therapeutic G-CSF support is initiated.

#### 6.4.3 Basic diagnostic work-up

Concurrently with G-CSF therapy, a diagnostic evaluation includes viral studies, exclusion of infections, ruling out IEC-HS (Immune Effector Cell-associated Hemophagocytic Syndrome), and exclusion of drug-induced causes.

#### 6.4.4 Extended work-up and BM biopsy for G-CSF refractory cases

If there is no count recovery despite ≥5 days of G-CSF support AND beyond day +14 post-CAR-T infusion, an extended diagnostic work-up including bone marrow biopsy, to identify underlying marrow pathology (aplasia, relapse, HLH/IEC-HS).

#### 6.4.5 Salvage therapies

For confirmed refractory ICAHT, two parallel rescue strategies are offered:

- Hematopoietic cell boost—autologous or allogeneic, if a cryopreserved graft is available
- TPO receptor agonists (romiplostim, eltrombopag) — can be used especially when thrombocytopenia co-exists, addressing megakaryocyte insufficiency, but evidence remains limited to case series and expert opinions, and prospective data are awaited.

#### 6.4.6 Allogeneic HSCT as ultima ratio

In carefully selected patients with severe, refractory aplasia and clinical deterioration despite the measures above, a donor search and allogeneic HSCT may be considered as a last-resort intervention. This approach is supported only by limited case-level experience, carries substantial risk in this heavily pre-treated population, and should not be regarded as a standard pathway.

## 7 Immune effector cell-associated HLH-like syndrome

Immune effector cell-associated hemophagocytic lymphohistiocytosis-like syndrome (IEC-HS) — also termed secondary HLH (sHLH) or macrophage activation syndrome (MAS) after cellular therapy — is a severe, potentially fatal complication characterised by extreme hypercytokinemia, hyperferritinemia, coagulopathy, multiorgan failure, and hemophagocytosis on bone marrow aspirate (26, 27). It represents the extremely severe end of the cytokine storm spectrum and overlaps clinically with Grade 3–4 CRS, but with additional hematological and coagulopathic features. Early recognition is crucial, as IEC-HS is associated with high mortality without intervention. IEC-HS must be distinguished from several overlapping entities. It should be emphasized that the diagnostic criteria applied to IEC-HS are adapted from sHLH standards and that current management rests largely on expert consensus and case-series data.

Diagnostic criteria adapted from EHA/EBMT guidelines for sHLH in the cellular therapies include: ferritin>10, 000 µg/L; evidence of hemophagocytosis on bone marrow aspirate; cytopenias in ≥2 lineages; elevated triglycerides; hypofibrinogenemia; and splenomegaly (26). Classical HLH is marked by the timing of cellular therapy and the lack of HLH-related genetic variants, although there are some similarities. Unlike severe CRS, with which it shares hypercytokinemia, IEC-HS characteristically emerges or worsens as CRS appears to be resolving and is defined by rapidly rising ferritin (often >10, 000 ug/L), hypofibrinogenemia, hypertriglyceridemia, progressive cytopenias, and hemophagocytosis. Sepsis must be excluded with cultures and clinical assessment, recognizing that procalcitonin is unreliable in this setting. Marrow morphology and immunophenotyping exclude disease progression with marrow infiltration. Management includes high-dose corticosteroids, anakinra (IL-1 blockade), tocilizumab, etoposide-based regimens in refractory cases, and ICU-level supportive care. Based on case series data, emapalumab (anti-IFN-γ antibody) can be considered as an emerging option in refractory IEC-HS (42, 43).

## 8 Infectious complications in the early phase

Infections represent a leading cause of non-relapse mortality after CAR-T cell therapy (27). The risk is a consequence of multiple concurrent immunosuppressive factors: lymphodepletion chemotherapy; CAR-T-induced B-cell aplasia and hypogammaglobulinemia; profound neutropenia and lymphopenia, and the prolonged use of corticosteroids for CRS/ICANS management. Recommendations should align with the actual immunological deficiency and time setting: neutropenia and mucosal injury dominate the first 30 days; B-cell aplasia with hypogammaglobulinaemia and delayed CD4 recovery dominate thereafter. In the first 30 days, bacterial infections predominate, including Gram-positive organisms (central line-associated) and Gram-negatives (gut translocation during neutropenia). Fungal infections, including invasive aspergillosis and candidiasis, are of particular concern during prolonged neutropenia (29, 44). Viral replication (CMV, EBV, HHV-6, BKV, and respiratory viruses) can be diagnosed in particular in patients with prior allogeneic HSCT. Pneumocystis jirovecii pneumonia can occur early if prophylaxis is inadequate. Management principles include risk-stratified antimicrobial prophylaxis, early empirical antibacterial therapy for febrile neutropenia, preemptive monitoring for CMV/EBV in high-risk patients, IVIG supplementation for IgG < 4 g/L during B-cell aplasia, and differential diagnosis between infection, CRS, and ICAHT-associated fever. Procalcitonin is unreliable during CRS — serial CRP and blood cultures remain the primary diagnostic tools (27). All patients should receive antiviral (acyclovir/valacyclovir) and PCP (co-trimoxazole) prophylaxis for at least 6 months and until CD4 count exceeds 200 cells/μL — noting that CD4 recovery may be significantly delayed with CD28ζ products and higher-intensity lymphodepletion. Antifungal coverage should be mould-active in high-risk patients (prior mould infection, allo-SCT, acute leukemia, grade ≥3 CRS/ICANS, or prolonged immunosuppression) and yeast-active with pre-emptive mould monitoring in standard-risk patients, continued until neutrophil recovery. Antibacterial fluoroquinolone prophylaxis remains controversial and should follow institutional guidelines with attention to local MDR-GNB prevalence.

## 9 Early complications of virus-specific T lymphocytes

### 9.1 Overview and product types

VSTs encompass a spectrum of T-cell products with specificity for viral antigens. In the post-HSCT context, the most clinically relevant products target EBV, CMV, adenovirus, BK virus, and HHV-6 (1). Products may be donor-derived (from the original HSCT donor), patient-derived (autologous, applicable in solid organ transplant recipients), or third-party off-the-shelf, partially HLA-matched from healthy seropositive donors (1, 4, 45). VSTs generated via cytokine capture systems (CliniMACS Cytokine Capture System; Miltenyi Biotec), short ex vivo expansion protocols, or clinical-grade lymphoblastoid cell line (LCL) stimulation protocols exhibit low rates of residual alloreactivity, as demonstrated in mixed lymphocyte reaction assays even across 3-antigen HLA mismatches (46–49).

**Figure 2:**
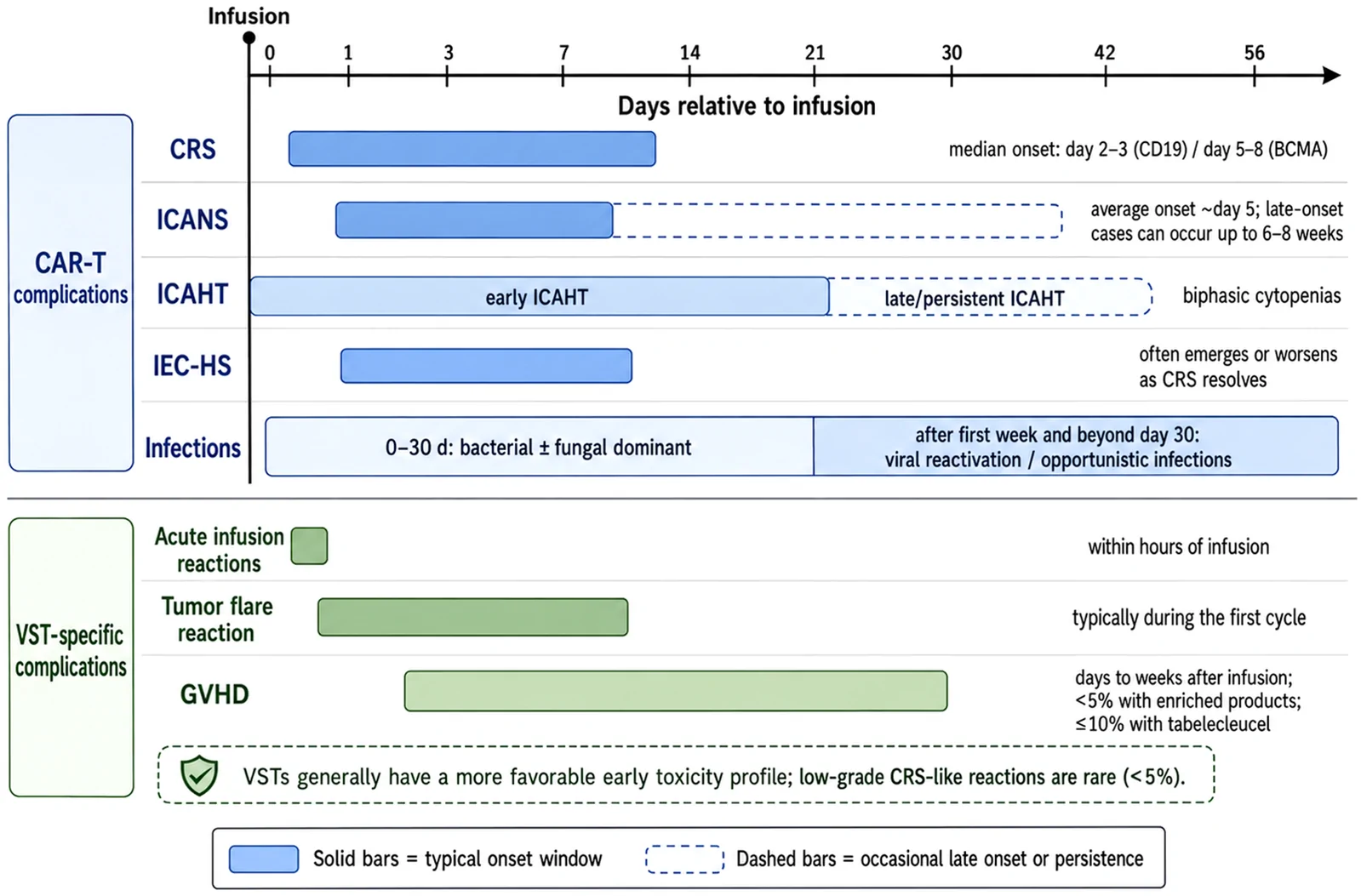
Typical early complications after cellular effector therapy.

The early toxicity profile of VSTs contrasts with that of CAR-T cell therapy, corresponding to a distinct mechanism of action: VSTs restore antiviral immunity via T cells with specific viral targeting, rather than inducing the extensive, antigen-driven effector proliferation connected to CRS, ICANS, and ICAHT following CAR-T infusion. As a result, the symptoms prevalent in the earlier sections are rare or nonexistent following VST treatment, with primary early concerns changing to tumor flare reaction, GVHD, acute infusion reactions, and sporadic low-grade CRS-like cytokine release (**Figure 2**).

**Table 5** summarizes major early toxicities; nevertheless, it must be interpreted with a caveat: the CAR-T data originate from large prospective trials, whereas the VST estimates are based on smaller, eclectic cohorts and regulatory documentation for tabelecleucel.

**Table 5:** Comparison of early toxicities: CAR-T versus VST.

Toxicity	CAR-T cell therapy	Virus-specific T lymphocytes
CRS	Common (37-95% any grade); onset 2–8 d	Uncommon, low-grade CRS (<5%); usually self-limiting; rarely needs tocilizumab
Neurotoxicity (ICANS)	20-60% any grade; product-dependent	Not characteristic of VSTs
Hematotoxicity (ICAHT)	Most frequent grade >=3 toxicity; biphasic	Not a defining feature; cytopenias relate to underlying disease/HSCT
Tumour flare reaction	Not typical	20% with tabelecleucel for EBV+ PTLD; days 1–14 post-infusion
GVHD	Not a feature of autologous products	<5% enriched/selected; <=10% tabelecleucel (SmPC)
Infusion reactions	Uncommon; mostly DMSO-related	2-5%; DMSO/debris-related
IEC-HS	Rare but severe	Not characteristic

### 9.2 Tumor flare reaction

Tumor flare reaction (TFR) is the most clinically important early complication specific to VST therapy for EBV+ PTLD, including patients receiving tabelecleucel. The EMA Summary of Product Characteristics (SmPC) identifies TFR as one of the most serious adverse reactions, occurring in approximately 20% of patients (4). TFR is defined as an immune activation reaction similar to worsening of the cancer, characterised by inflammation at tumor sites. Clinically, TFR presents with rapid enlargement of lymph nodes or other tumor masses, generalized symptoms (fever, bone pain, skin rash), and elevated inflammatory markers. It typically occurs within the first 1–2 cycles of treatment (predominantly days 1–14 after infusion). The critical diagnostic challenge is distinguishing TFR from true disease progression, which requires repeat imaging (PET-CT or CT) and clinical correlation: TFR may benefit from continuation of therapy with appropriate symptomatic management, while genuine progression warrants treatment modification. EBV DNA may transiently rise during TFR before declining with therapeutic response, consistent with immune-mediated tumor destruction (50).

Management of TFR includes: anti-pyretics and NSAIDs for symptom relief; short-course corticosteroids (dexamethasone 4–8 mg daily for 5–7 days) for significant symptomatic reactions, analgesics for bone pain, and close monitoring for airway compromise in cases of rapid cervical lymphadenopathy (4).

### 9.3 Graft-versus-host disease

Graft-versus-host disease (GVHD) is the primary safety concern for allogeneic VST administration and is driven by alloreactive T cells within the infused product targeting host tissues (2, 3). Early studies using unmanipulated donor lymphocyte infusions (DLI) for EBV-PTLD reported GVHD rates of 10–15%, significantly limiting their application in the early post-HSCT period. Enrichment and selection of VSTs — via IFN-γ capture, MACS selection, or rapid expansion protocols — has reduced alloreactivity to consistently below 5% in large published series (1).

For tabelecleucel SmPC specifically, GVHD is listed as a serious adverse reaction that may affect up to 1 in 10 people (<=10%) (4). First-line treatment is corticosteroids, with careful reduction of concurrent immunosuppression to avoid exacerbating underlying PTLD. Clinical vigilance for classic acute GVHD manifestations (cutaneous rash, gastrointestinal symptoms, elevated liver transaminases, and bilirubin) is recommended from day 1 onward.

### 9.4 Infusion reactions and CRS-like toxicity

Acute infusion reactions occur in approximately 2–5% of VSTs infusions. Manifestations include rigors, fever, urticaria, hypotension, and bronchospasm, often attributed to cryoprotectants (DMSO) or residual cellular debris in the cryopreserved and thawed VST product. Pre-medication with antihistamine (chlorphenamine 10 mg IV) and hydrocortisone (100 mg IV) 30–60 minutes before infusion is recommended by institutional protocols.

Low-grade CRS-like cytokine release syndrome (Grade 1–2) has been reported following VST infusions, particularly in patients with high EBV tumor burdens. The pathophysiology involves IL-6, IFN-γ, and TNF-α release upon T-cell activation at tumor sites. Management follows standard CRS grading principles; tocilizumab is rarely required for VST-associated CRS given its typically mild and self-limiting nature.

### 9.5 VST complication summary

The early complications of VST therapy reflect the lower alloreactivity and more physiological activation of virus-specific products. **Table 6** summarizes these complications—graft-versus-host disease, tumor flare reaction, low-grade CRS-like cytokine release, and acute infusion reactions—with their approximate incidence, mechanism, and management. The evidence base is less robust than for CAR-T cells, given the heterogeneity of products, manufacturing platforms, and patient populations across the underlying studies, and the reported rates should be interpreted as suggestive rather than directly comparable.

**Table 6:** Early complications of virus-specific T lymphocytes.

Complication	Incidence	Pathophysiology	Management
Graft-versus-Host Disease (GVHD)	< 5% with enriched VSTs; ≤ 10% with tabelecleucel.	Alloreactive donor T cells in infused product; higher risk with HLA-mismatched donors	Corticosteroids; careful adjustment of immunosuppression; tacrolimus
Tumor Flare Reaction (TFR)	20% for EBV-VST/PTLD, including tabelecleucel; typically days 1–14 of the 1st cycle	Immune activation and local inflammation at tumor sites. Lymph node enlargement, fever	Antipyretics, NSAIDs, and short-course corticosteroids (dexamethasone 4–8 mg × 5–7 days); continue therapy if manageable.
CRS-like reaction	< 5% of VST infusions; generally grade 1–2	Rapid T-cell expansion; cytokine surge (IL-6, IFN-γ, TNF-α)	Supportive; tocilizumab for grade ≥2; antipyretics
Acute Infusion Reactions	2–5%; within hours of infusion	Immediate hypersensitivity; DMSO cryoprotectant toxicity	Pre-medication (antihistaminics + hydrocortisone 30–60 min pre-infusion); reduce infusion rate

## 10 Clinical monitoring protocol

Clinical monitoring is fundamental to early detection and pre-emptive management of complications in cellular therapy recipients (**Table 7**). The EBMT/EHA CAR-T Cell Handbook and EBMT Immune Effector Cell Nursing Guidelines require a minimum of 14 days of inpatient monitoring after CAR-T infusion (30). Inpatient monitoring for roughly 10–14 days after a CAR-T infusion is a common and conservative practice, reflecting that most grade >=3 CRS and ICANS events occur within this window. It is not an absolute requirement: many centers now use product-specific and risk-adapted pathways, managing lower-toxicity products in outpatient or day-hospital settings while extending inpatient observation for high tumor burden, high CAR-HEMATOTOX score, or early toxicity. Irrespective of inpatient duration, patients are generally advised to remain close to the treating center with a caregiver for approximately four weeks given the risk of late ICANS.

**Table 7:** Recommended clinical monitoring schedule after cellular therapy infusion.

Timepoint	Clinical assessment	Laboratory tests	Imaging
Pre-infusion baseline	Vital signs; ECOG PS; neurological exam (ICE score); weight	CBC, CMP, coagulation, CRP, ferritin, fibrinogen, LDH, IL-6	PET-CT (disease burden); echo if cardiac risk
Day 0–7 (post-infusion)	Vitals q4–6h; neurological screening q8–12h (ICE test, handwriting); fever monitoring	Daily CBC; CRP/ferritin daily; coagulation; hepatic/renal panel	Brain MRI if ICANS ≥ G2 or new neurological signs
Day 7–14	Continued vitals; neurological exam; ICAHT assessment	CBC 3×/week; ferritin/CRP 2×/week; LDH	CT if tumor flare is suspected
Day 14–30	Clinical review; response assessment	Weekly CBC; LFTs; ferritin if cytopenias persist; immunoglobulin levels	Response/staging around day 28-30 (VST/CAR-T)

ICE, Immune Effector Cell-Associated Encephalopathy score; ECOG PS, Eastern Cooperative Oncology Group Performance Status.

VST recipients may be managed in outpatient or day-hospital settings depending on disease burden and patient risk profile, though the tabelecleucel SmPC mandates access to facilities capable of managing severe adverse reactions, including those requiring urgent interventions.

Key surveillance points include ICE screening every 8–12 hours during the first 7 days; handwriting test at each neurological assessment; temperature monitoring every 4 hours; and continuous pulse oximetry for Grade ≥1 CRS. Any deterioration in neurological status, new fever, or cardiorespiratory instability must be escalated immediately to the medical team.

## 11 ICU escalation criteria

The EBMT/EHA Handbook dedicates a specific chapter to ICU management of CAR-T recipients, recognizing that the spectrum of toxicity may rapidly progress to life-threatening hemodynamic instability, respiratory failure, or refractory neurological dysfunction (27).

Absolute criteria for ICU admission:

- Grade 3 or 4 CRS with hemodynamic instability requiring vasopressors
- Oxygen requirement > 6 L/min nasal cannula or > 40% FiO_2_
- Grade 4 ICANS: coma, status epilepticus, evidence of cerebral edema
- Active seizures not responding to first-line anticonvulsants
- IEC-HS with multiorgan dysfunction
- Requirement for mechanical ventilation
- New cardiac dysfunction (arrhythmia, myocarditis, cardiogenic shock)

Relative criteria requiring intermediate care:

- Grade 2 CRS with significant respiratory compromise or oliguria
- Grade 3 ICANS with evolving obtundation
- Rapidly deteriorating laboratory parameters (ferritin > 10, 000 µg/L, worsening coagulopathy)
- Comorbidities increase the risk of rapid decompensation.

## 12 Special considerations in pediatric patients

Tabelecleucel is approved in pediatric patients from 2 years of age, and CAR-T tisagenlecleucel is approved for pediatric B-ALL from 3 years of age. Clinical management of early complications in children requires adaptation of grading tools, age-appropriate communication, and recognition of distinct developmental vulnerabilities (51). In pediatric patients receiving CAR-T cell therapy, standard ICANS assessment using the ICE score may be developmentally inappropriate or impossible to apply, particularly in younger children, infants, and those with pre-existing neurodevelopmental conditions. Clinical presentation in young children may be atypical, including irritability, abnormal behavior, and regression of developmental skills as early signs. The Cornell Assessment of Pediatric Delirium (CAPD) is a validated eight-item observational tool applicable from birth to 21 years, completed by the bedside nurse in under two minutes and requiring no patient cooperation (52). A total score of ≥9 indicates a high probability of delirium and should prompt further clinical evaluation and consideration of intervention. The CAPD should not be applied in children with a Richmond Agitation and Sedation Scale (RASS) score below −3, as deep sedation or complete unresponsiveness precludes reliable behavioral observation.

Equally important is psychosocial support, which should include age-appropriate explanations of treatment effects, the use of play therapy when appropriate, and involvement of child life specialists, with family-centered care remaining a fundamental component of management.

Finally, in pediatric practice, drug dosing should be adapted to body weight. Tocilizumab is administered at 12 mg/kg in children weighing less than 30 kg and at 8 mg/kg intravenously in those weighing 30 kg or more, with a maximum dose of 800 mg, while dexamethasone should likewise be adjusted according to body weight.

## 13 Distinguishing overlapping syndromes

A major clinical challenge in cellular therapy is the simultaneous or sequential occurrence of multiple toxicity syndromes, combined with the risk of infectious complications that closely mimic CRS. The following diagnostic approaches aid clinical differentiation:

- CRS vs. Sepsis: Both present with fever, hypotension, and elevated inflammatory markers.

Key differentiators: CRS onset typically 2–14 days post-infusion; markedly elevated ferritin; procalcitonin has limited utility in neutropenic patients; blood cultures mandatory in all febrile post-infusion patients; empirical antibiotics must not be withheld pending cytokine results.

- ICANS vs. CNS Infection: ICANS is a clinical diagnosis; MRI and CSF may be normal or non-specifically abnormal. CSF PCR for HSV, CMV, EBV, HHV-6, and VZV must be obtained if clinical uncertainty exists. ICANS typically co-occurs with or follows CRS; isolated early CNS events without CRS should prompt expanded infection workup.
- Tumor Flare Reaction vs. Disease progression (VSTs): Both present with lymphadenopathy and constitutional symptoms.

Key differentiators: TFR typically occurs earlier (days 1–14) and is associated with inflammatory markers; serial PET-CT with SUV quantification; absence of new extranodal sites in TFR; transient EBV DNA rise distinguishes TFR from progression.

- ICAHT vs. Disease relapse with marrow infiltration: Both cause pancytopenia after CAR-T. Bone marrow aspirate and biopsy is essential; immunophenotyping distinguishes residual/relapsed tumor from aplastic marrow; CAR-T PCR in marrow confirms T-cell persistence.
- IEC-HS vs. Severe CRS: Both share hypercytokinemia and organ dysfunction. Key differentiators: IEC-HS distinguished by ferritin >10, 000 µg/L, hemophagocytosis, hypofibrinogenemia, hypertriglyceridemia, and splenomegaly.

## 14 Summary and conclusions

Immunotherapies with effector cells have established clinical efficacy in hematological malignancies and post-transplant viral diseases. Their toxicity profiles are distinct, well-characterised, and manageable with institutional protocols aligned to international guidelines. For CAR-T cell therapy, CRS and ICANS remain the primary early complications requiring immediate clinical attention. For VSTs, the early safety profile is considerably more favorable. Tumor flare reaction is the most clinically relevant early complication, GVHD though rare with selected products, remains a priority given its potentially severe consequences in already immunosuppressed transplant recipients. The growing body of real-world experience with CAR-T cell therapy has translated into increasingly standardized, evidence-informed approaches to toxicity recognition and management beyond the controlled trial setting (53). The ongoing EBVOLVE study for tabelecleucel will help better assess risks and guide future updates to guidelines by adding to real-world EBMT registries for CAR-T products and improving predictions of side effects.

## Glossary

AdV: Adenovirus
allo-HCT: Allogeneic Hematopoietic Cell Transplantation
ASTCT: American Society for Transplantation and Cellular Therapy
ATMP: Advanced Therapy Medicinal Product
B-ALL: B-cell Acute Lymphoblastic Leukemia
BCMA: B-Cell Maturation Antigen
BKV: BK Polyomavirus
CAR: Chimeric Antigen Receptor
CBC: complete blood counts
CMV: Cytomegalovirus
CRS: Cytokine Release Syndrome
CSF: Cerebrospinal Fluid
CTCAE: Common Terminology Criteria for Adverse Events
DLI: Donor Lymphocyte Infusion
DMSO: dimethylosulfoxide
EBMT: European Society for Blood and Marrow Transplantation
EBV: Epstein-Barr Virus
EHA: European Hematology Association
EMA: European Medicines Agency
G-CSF: Granulocyte Colony-Stimulating Factor
GVHD: Graft-versus-Host Disease
HHV-6: Human Herpesvirus 6
HLH: Hemophagocytic Lymphohistiocytosis
HSCT: Hematopoietic Stem Cell Transplantation
ICE: Immune Effector Cell-Associated Encephalopathy
ICANS: Immune Effector Cell-Associated Neurotoxicity Syndrome
ICAHT: Immune Effector Cell-Associated Hematotoxicity
IEC-HS: Immune Effector Cell-Associated HLH-like Syndrome
IL: Interleukin
LBCL: Large B-Cell Lymphoma
LFT: liver function test
MAS: Macrophage Activation Syndrome
MCL: Mantle Cell Lymphoma
MM: Multiple Myeloma
NRM: Non-Relapse Mortality
PTLD: Post-Transplant Lymphoproliferative Disease
sHLH: Secondary HLH
TFR: Tumor Flare Reaction
TPO-RA: Thrombopoietin Receptor Agonist
VST: Virus-Specific T Lymphocyte.

## References

[1] Koukoulias K Papayanni PG Leen AM Vasileiou S . Virus-specific T-cell therapy for the management of viral infections in the immunocompromised. Transfusion Med Hemotherapy. (2024) 2024:1–22. doi: 10.1159/000540961 39944414 PMC11813280

[2] Haque T Wilkie GM Jones MM Higgins CD Urquhart G Wingate P . Allogeneic cytotoxic T-cell therapy for EBV-positive posttransplantation lymphoproliferative disease: results of a phase 2 multicenter clinical trial. Blood. (2007) 110:1123–31. doi: 10.1182/blood-2006-12-063008 17468341

[3] Rooney CM Smith CA Ng CYC Loftin SK Sixbey JW Gan Y . Infusion of cytotoxic T cells for the prevention and treatment of Epstein-Barr virus-induced lymphoma in allogeneic transplant recipients. Blood. (1998) 92:1549–55. doi: 10.1182/blood.V92.5.1549 9716582

[4] Ebvallo: EPAR - Product Information. Summary of Product Characteristics (Smpc). Available online at: https://www.ema.europa.eu/en/documents/product-information/ebvallo-epar-product-information_en.pdf (Accessed January 15, 2026).

[5] Yakoub-Agha I Chabannon C Bader P Basak GW Bonig H Ciceri F . Management of adults and children undergoing chimeric antigen receptor T-cell therapy: best practice recommendations of the European Society for Blood and Marrow Transplantation (EBMT) and the Joint Accreditation Committee of ISCT and EBMT (JACIE). Haematologica. (2020) 105:297–316. doi: 10.3324/haematol.2019.229781 31753925 PMC7012497

[6] Lee DW Santomasso BD Locke FL Ghobadi A Turtle CJ Brudno JN . ASTCT consensus grading for cytokine release syndrome and neurologic toxicity associated with immune effector cells. Biol Blood Marrow Transplant. (2019) 25:625–38. doi: 10.1016/j.bbmt.2018.12.758 30592986 PMC12180426

[7] Thompson JA Schneider BJ Brahmer J Achufusi A Armand P Berkenstock MK . Management of immunotherapy-related toxicities, version 1.2022, NCCN clinical practice guidelines in oncology. J Natl Compr Cancer Network. (2022) 20:387–405. doi: 10.6004/jnccn.2022.0020 35390769

[8] Neelapu SS Locke FL Bartlett NL Lekakis LJ Miklos DB Jacobson CA . Axicabtagene ciloleucel CAR T-cell therapy in refractory large B-cell lymphoma. N Engl J Med. (2017) 377:2531–44. doi: 10.1056/NEJMoa1707447 29226797 PMC5882485

[9] Schuster SJ Bishop MR Tam CS Waller EK Borchmann P McGuirk JP . Tisagenlecleucel in adult relapsed or refractory diffuse large B-cell lymphoma. N Engl J Med. (2019) 380:45–56. doi: 10.1056/NEJMoa1804980 30501490

[10] van Meerten T Kersten MJ Iacoboni G Hess GR Mutsaers PGNJ Martin Garcia-Sancho AM . Brexucabtagene autoleucel for BTKi-naive relapsed/refractory mantle cell lymphoma: primary analysis of ZUMA-2 Cohort 3. Blood. (2026) 147:1302–14. doi: 10.1182/blood.2025029734 41160777 PMC13014085

[11] Yared JA Fromowitz A Kocoglu M Hardy N Atanackovic D Rapoport AP . Obecabtagene autoleucel, a novel CD19-directed CAR T-cell therapy for relapsed/refractory B-cell acute lymphoblastic leukemia: the future for reducing toxicity and T-cell exhaustion? Expert Rev Hematol. (2025) 18:585–93. doi: 10.1080/17474086.2025.2523551 40550480

[12] Roloff GW Aldoss I Kopmar NE Lin C Dekker SE Gupta VK . Outcomes after brexucabtagene autoleucel administered as a standard therapy for adults with relapsed/refractory B-cell ALL. J Clin Oncol. (2025) 43:558–66. doi: 10.1200/JCO.24.00321 39418622 PMC12070553

[13] Roddie C Sandhu KS Tholouli E Logan AC Shaughnessy P Barba P . Obecabtagene autoleucel in adults with B-cell acute lymphoblastic leukemia. N Engl J Med. (2024) 391:2219–30. doi: 10.1056/NEJMoa2406526 39602653 PMC12818175

[14] Maude SL Laetsch TW Buechner J Rives S Boyer M Bittencourt H . Tisagenlecleucel in children and young adults with B-cell lymphoblastic leukemia. N Engl J Med. (2018) 378:439–48. doi: 10.1056/NEJMoa1709866 29385370 PMC5996391

[15] Abramson JS Palomba ML Gordon LI Lunning MA Wang M Arnason J . Lisocabtagene maraleucel for patients with relapsed or refractory large B-cell lymphomas (TRANSCEND NHL 001): a multicentre seamless design study. Lancet. (2020) 396:839–52. doi: 10.1016/S0140-6736(20)31366-0 32888407

[16] Shah BD Ghobadi A Oluwole OO Logan AC Boissel N Cassaday RD . KTE-X19 for relapsed or refractory adult B-cell acute lymphoblastic leukaemia: phase 2 results of the single-arm, open-label, multicentre ZUMA-3 study. Lancet. (2021) 398:491–502. doi: 10.1016/S0140-6736(21)01222-8 34097852 PMC11613962

[17] Munshi NC Anderson LD Shah N Madduri D Berdeja J Lonial S . Idecabtagene vicleucel in relapsed and refractory multiple myeloma. N Engl J Med. (2021) 384:705–16. doi: 10.1056/NEJMoa2024850 33626253

[18] Berdeja JG Madduri D Usmani SZ Jakubowiak A Agha M Cohen AD . Ciltacabtagene autoleucel, a B-cell maturation antigen-directed chimeric antigen receptor T-cell therapy in patients with relapsed or refractory multiple myeloma (CARTITUDE-1): a phase 1b/2 open-label study. Lancet. (2021) 398:314–24. doi: 10.1016/S0140-6736(21)00933-8 34175021

[19] An Observational, Post-Authorisation Safety Study (PASS) to Describe the Safety and Effectiveness of Tabelecleucel in Patients With Epstein-Barr Virus Positive (EBV+) Post-Transplant Lymphoproliferative Disease (PTLD) in a Real-World Setting in Europe: EBVOLVE Study [EUPAS1000000113]. Available online at: https://catalogues.ema.europa.eu/node/4014/administrative-details (Accessed February 28, 2026).

[20] Mahadeo KM Baiocchi R Beitinjaneh A Chaganti S Choquet S Dierickx D . Tabelecleucel for allogeneic haematopoietic stem-cell or solid organ transplant recipients with Epstein–Barr virus-positive post-transplant lymphoproliferative disease after failure of rituximab or rituximab and chemotherapy (ALLELE): a phase 3, multicentre, open-label trial. Lancet Oncol. (2024) 25:376–87. doi: 10.1016/S1470-2045(23)00649-6 38309282

[21] Ayuketang FA Jäger U Sanderson R Chiappella A . 40 - management of CRS and IEC-associated haemophagocytic lymphohistiocytosis-like syndrome (IEC-HS), in: The Eu Car-T Handbook. Available online at: https://www.ebmt.org/education/carthandbook/ (Accessed February 26, 2026).

[22] Rees J Velasco R . 41 - management of IEC-associated neurotoxicity syndrome (ICANS) and non-ICANS neurotoxicity, in: The Eu Car-T Handbook. Available online at: https://www.ebmt.org/education/carthandbook/ (Accessed February 27, 2026).

[23] Rejeski K Benjamin R . 42 - Management of ICAHT, Infectious Complications, Hypogammaglobulinemia and B cell Aplasia, in: The Eu Car-T Handbook. Available online at: https://www.ebmt.org/education/carthandbook/ (Accessed February 27, 2026).

[24] Gust J Hay KA Hanafi L-A Li D Myerson D Gonzalez-Cuyar LF . Endothelial activation and blood–brain barrier disruption in neurotoxicity after adoptive immunotherapy with CD19 CAR-T cells. Cancer Discov. (2017) 7:1404–19. doi: 10.1158/2159-8290.CD-17-0698 29025771 PMC5718945

[25] Santomasso BD Park JH Salloum D Riviere I Flynn J Mead E . Clinical and biological correlates of neurotoxicity associated with CAR T-cell therapy in patients with B-cell acute lymphoblastic leukemia. Cancer Discov. (2018) 8:958–71. doi: 10.1158/2159-8290.CD-17-1319 29880584 PMC6385599

[26] Rejeski K Subklewe M Aljurf M Bachy E Balduzzi A Barba P . Immune effector cell–associated hematotoxicity: EHA/EBMT consensus grading and best practice recommendations. Blood. (2023) 142:865–77. doi: 10.1182/blood.2023020578 37300386

[27] Kröger N Gribben J Chabannon C Yakoub-Agha I Einsele H . The Ebmt/Eha CAR-T Cell Handbook. Cham: Springer International Publishing (2022). doi: 10.1007/978-3-030-94353-0 36121969

[28] Rejeski K Wang Y Hansen DK Iacoboni G Bachy E Bansal R . Applying the EHA/EBMT grading for ICAHT after CAR-T: comparative incidence and association with infections and mortality. Blood Adv. (2024) 8:1857–68. doi: 10.1182/bloodadvances.2023011767 38181508 PMC11007437

[29] Taplitz RA Kennedy EB Bow EJ Crews J Gleason C Hawley DK . Antimicrobial prophylaxis for adult patients with cancer-related immunosuppression: ASCO and IDSA clinical practice guideline update. J Clin Oncol. (2018) 36:3043–54. doi: 10.1200/JCO.18.00374 30179565

[30] Ellard R Kenyon M Hutt D Aerts E de Ruijter M Chabannon C . The EBMT immune effector cell nursing guidelines on CAR-T therapy: a framework for patient care and managing common toxicities. Clin Hematol Int. (2022) 4:75–88. doi: 10.1007/s44228-022-00004-8 36131128 PMC9263804

[31] Le RQ Li L Yuan W Shord SS Nie L Habtemariam BA . FDA approval summary: Tocilizumab for treatment of chimeric antigen receptor T cell-induced severe or life-threatening cytokine release syndrome. Oncologist. (2018) 23:943–7. doi: 10.1634/theoncologist.2018-0028 29622697 PMC6156173

[32] Locke FL Neelapu SS Bartlett NL Lekakis LJ Jacobson CA Braunschweig I . Tocilizumab prophylaxis following axicabtagene ciloleucel in relapsed or refractory large B-cell lymphoma. Transplant Cell Ther. (2024) 30:1065–79. doi: 10.1016/j.jtct.2024.08.018 39187161

[33] Wang WL Lee D Cheung E Jensen A Hamilton MP Rana MS . Early steroid and anakinra use to manage axicabtagene ciloleucel toxicity reduces the total duration of CRS and ICANS. Transplant Cell Ther. (2025) 31:S138–9. doi: 10.1016/j.jtct.2025.01.211 41855506 PMC13194643

[34] Neill L Rees J Roddie C . Neurotoxicity—CAR T-cell therapy: what the neurologist needs to know. Pract Neurol. (2020) 20:285–93. doi: 10.1136/practneurol-2020-002550 32503897

[35] Gazeau N Liang EC Wu Q“ Voutsinas JM Barba P Iacoboni G . Anakinra for refractory cytokine release syndrome or immune effector cell-associated neurotoxicity syndrome after chimeric antigen receptor T cell therapy. Transplant Cell Ther. (2023) 29:430–7. doi: 10.1016/j.jtct.2023.04.001 37031746 PMC10330552

[36] Shah NN Johnson BD Fenske TS Raj RV Hari P . Intrathecal chemotherapy for management of steroid-refractory CAR T-cell–associated neurotoxicity syndrome. Blood Adv. (2020) 4:2119–22. doi: 10.1182/bloodadvances.2020001626 32407473 PMC7252557

[37] Bashey SZ Solomon SR Zhang X Morris LE Holland HK Bachier L . Intrathecal chemotherapy as treatment for chimeric antigen receptor T cell (CAR T) therapy associated neurotoxicity. Bone Marrow Transplant. (2024) 59:1783–5. doi: 10.1038/s41409-024-02417-w 39300248

[38] Rejeski K Jain MD Shah NN Perales M-A Subklewe M . Immune effector cell-associated haematotoxicity after CAR T-cell therapy: from mechanism to management. Lancet Haematol. (2024) 11:e459–70. doi: 10.1016/S2352-3026(24)00077-2 38734026 PMC12413773

[39] Rejeski K Hansen DK Bansal R Sesques P Ailawadhi S Logue JM . The CAR-HEMATOTOX score as a prognostic model of toxicity and response in patients receiving BCMA-directed CAR-T for relapsed/refractory multiple myeloma. J Hematol Oncol. (2023) 16:88. doi: 10.1186/s13045-023-01465-x 37525244 PMC10391746

[40] Nair MS Silbert SK Rejeski K Wilson KA Lamble AJ Valtis Y . Development of ALL-Hematotox: predicting post-CAR T-cell hematotoxicity in B-cell acute lymphoblastic leukemia. Blood. (2025) 145:1136–48. doi: 10.1182/blood.2024025910 39561284 PMC11923441

[41] Miller KC Johnson PC Abramson JS Soumerai JD Yee AJ Branagan AR . Effect of granulocyte colony-stimulating factor on toxicities after CAR T cell therapy for lymphoma and myeloma. Blood Cancer J. (2022) 12:146. doi: 10.1038/s41408-022-00741-2 36316312 PMC9622902

[42] McNerney KO Diorio C Annesley C Gardner RA Graham AK Sanchez-Pinto LN . Management practices of CAR T-cell-related inflammatory toxicities: a survey of pediatric CAR T-cell providers. Transplant Cell Ther. (2026) 32:158–68. doi: 10.1016/j.jtct.2025.09.028 41038347 PMC12823210

[43] Manni S Del Bufalo F Merli P Silvestris DA Guercio M Caruso S . Neutralizing IFNγ improves safety without compromising efficacy of CAR-T cell therapy in B-cell Malignancies. Nat Commun. (2023) 14:3423. doi: 10.1038/s41467-023-38723-y 37296093 PMC10256701

[44] Shahid Z Jain T Dioverti V Pennisi M Mikkilineni L Thiruvengadam SK . Best practice considerations by the American Society of Transplant and Cellular Therapy: infection prevention and management after chimeric antigen receptor T cell therapy for hematological Malignancies. Transplant Cell Ther. (2024) 30:955–69. doi: 10.1016/j.jtct.2024.07.018 39084261 PMC12826105

[45] Tzannou I Papadopoulou A Naik S Leung K Martinez CA Ramos CA . Off-the-shelf virus-specific T cells to treat BK virus, human herpesvirus 6, cytomegalovirus, Epstein-Barr virus, and adenovirus infections after allogeneic hematopoietic stem-cell transplantation. J Clin Oncol. (2017) 35:3547–57. doi: 10.1200/JCO.2017.73.0655 28783452 PMC5662844

[46] Kaeuferle T Krauss R Blaeschke F Willier S Feuchtinger T . Strategies of adoptive T-cell transfer to treat refractory viral infections post allogeneic stem cell transplantation. J Hematol Oncol. (2019) 12:13. doi: 10.1186/s13045-019-0701-1 30728058 PMC6364410

[47] Feuchtinger T Lang P Hamprecht K Schumm M Greil J Jahn G . Isolation and expansion of human adenovirus-specific CD4+ and CD8+ T cells according to IFN-γ secretion for adjuvant immunotherapy. Exp Hematol. (2004) 32:282–9. doi: 10.1016/j.exphem.2003.12.009 15003314

[48] Feuchtinger T Matthes‐Martin S Richard C Lion T Fuhrer M Hamprecht K . Safe adoptive transfer of virus‐specific T‐cell immunity for the treatment of systemic adenovirus infection after allogeneic stem cell transplantation. Br J Haematol. (2006) 134:64–76. doi: 10.1111/j.1365-2141.2006.06108.x 16803570

[49] Heinz AT Calkoen FGJ Derbich A Miltner L Seitz C Doering M . Automated production of specific T cells for treatment of refractory viral infections after allogeneic stem cell transplantation. Haematologica. (2023) 108:2080–90. doi: 10.3324/haematol.2022.281996 36794500 PMC10388273

[50] Styczynski J van der Velden W Fox CP Engelhard D de la Camara R Cordonnier C . Management of Epstein-Barr virus infections and post-transplant lymphoproliferative disorders in patients after allogeneic hematopoietic stem cell transplantation: Sixth European Conference on Infections in Leukemia (ECIL-6) guidelines. Haematologica. (2016) 101:803–11. doi: 10.3324/haematol.2016.144428 27365460 PMC5004459

[51] Falkenburg JHF Schmid C Kolb HJ Kuball J . The Ebmt Handbook. Sureda A Corbacioglu S Greco R Kröger N Carreras E , editors. Cham: Springer International Publishing (2024) p. 531–9. doi: 10.1007/978-3-031-44080-9

[52] Traube C Silver G Kearney J Patel A Atkinson TM Yoon MJ . Cornell assessment of pediatric delirium. Crit Care Med. (2014) 42:656–63. doi: 10.1097/CCM.0b013e3182a66b76 24145848 PMC5527829

[53] Jain MD Smith M Shah NN . How I treat refractory CRS and ICANS after CAR T-cell therapy. Blood. (2023) 141:2430–42. doi: 10.1182/blood.2022017414 36989488 PMC10329191

